# PCR Diagnosis of Small Hive Beetles

**DOI:** 10.3390/insects9010024

**Published:** 2018-02-14

**Authors:** Franck Ouessou Idrissou, Qiang Huang, Orlando Yañez, Kayode Lawrence Akinwande, Peter Neumann

**Affiliations:** 1 Institute of Bee Health, Vetsuisse Faculty, University of Bern, 3003 Bern, Switzerland; qiang.huang@vetsuisse.unibe.ch (Q.H.); orlando.yanez@vetsuisse.unibe.ch (O.Y.); kayode.akinwande@vetsuisse.unibe.ch (K.L.A.); peter.neumann@vetsuisse.unibe.ch (P.N.); 2 Agroscope, Swiss Bee Research Centre, 3003 Bern, Switzerland; 3 Department of Biology, Federal University of Technology, P.M.B. 704 Akure, Nigeria

**Keywords:** *Aethina tumida*, *Apis mellifera*, honey bee

## Abstract

Small hive beetles (SHBs), *Aethina tumida*, are parasites of social bee colonies native to sub-Saharan Africa and have become an invasive species at a global scale. Reliable Polymerase Chain Reaction (PCR) diagnosis of this mandatory pest is required to limit its further spread and impact. Here, we have developed SHB primers, which amplify for 10 native African locations and 10 reported introductions, but not for three closely related species (*Aethina concolor*, *Aethina flavicollis*, and *Aethina inconspicua*). We also show that adult honey bee workers can be used as matrices for PCR-based detection of SHBs. The sensitivity of this novel method appears to be 100%, which is identical to conventional visual screenings. Furthermore, the specificity of this novel approach was also high (90.91%). Since both sensitivity and specificity are high, we recommend this novel PCR method and the new primers for routine surveillance of hives in high-risk areas.

## 1. Introduction

Small hive beetles (SHBs), *Aethina tumida* Murray (Coleoptera: Nitidulidae), are parasites of social bee colonies native to sub-Saharan Africa [1]. Larval and adult *A. tumida* feed on honey, pollen, bee larvae, as well as dead adult bees [1,2,3]. Feeding larvae can cause severe damage to combs of both African and European honey bee colonies, *Apis mellifera* Linnaeus (Hymenoptera: Apidae, [4]), which can result in the full structural collapse of the nest [5].

Recently, SHBs have emerged as an invasive species and were first noticed in November 1996 in South Carolina in the United States [1]. Since then, they have spread rapidly and been recorded from Egypt (2000), Australia (2001), Canada (2002), Portugal (2004), Jamaica (2005), Mexico (2007), Hawaii (2010), Cuba (2012), El Salvador (2013), Nicaragua (2014), Italy (2014), the Philippines (2014), Brazil (2016), and South Korea (2017) [1,6,7]. In its new ranges, SHBs can have a strong impact on honey bees [4] and can also infest colonies of other social bees [1].

Many countries have now included measures to slow down the spread of the SHB [1,8]. This includes quarantine measures, as well as diagnosis of colonies [1,8]. However, morphometric diagnosis is hampered by similarities with other beetle species associated with bee colonies [1]. This creates a demand for a reliable species-specific PCR. However, previous primers for SHB detection [9,10,11] are not able to detect all SHB populations (e.g., Ethiopian, unpublished data) and therefore bear the risk of false negative results. Moreover, due to frequent and close interaction between SHBs and adult honey bee workers (trophallaxis, aggression, etc., [1]), it appears very plausible that hosts will receive SHB DNA. Given that holds true, adult honey bee workers could serve as matrices for PCR-based diagnostics. Here, we developed novel species-specific primers for identification of *A. tumida* and tested whether SHB DNA can be detected on adult workers of infested and non-infested honey bee colonies.

## 2. Materials and Methods

### 2.1. Sample Collection

Adult SHBs were sampled from 20 infested *Apis mellifera* colonies from 10 native locations in Africa (Benin, Togo, Nigeria, Central African Republic, Burkina Faso, Democratic Republic of Congo, South Africa, Ethiopia, Tanzania, and Madagascar) and from 10 SHB introductions (US-Lousiana1, US-Louisiana2, Brazil, Costa-Rica, Australia-Cairns, Australia-Nambour, Portugal, Italy, Jamaica, and South Korea). *Aethina concolor*, *Aethina flavicollis*, and *Aethina inconspicua* specimens were sampled in Australia and South Korea. All *Aethina* samples were preserved in 70% Ethanol, transported at room temperature room temperature room temperature and stored at −20 °C in the laboratory until further analyses. All *Aethina* sampling sites covered the natural range of *A. m. adansonii*, *A. m. capensis*, *A. m. scutellata*, *A. m. unicolor*, *A. m. simensis*, *A. m. ligustica*, *A. m. iberiensis*, and *A. m. mellifera*.

In Nigeria, 100 adult honey bee workers (*A. m. adansonii*) per colony were sampled from SHB infested colonies (*N* = 10) and non-infested colonies (*N* = 10) by shaking brood frames into envelopes. SHB infestation status of colonies was evaluated by visual screening of bottom boards [11]. Similarly, in Switzerland (SHB-free country), 100 adult honey bee workers (*A. m. carnica*) were also sampled from 10 colonies at two apiaries (Aare and Liebefeld). These latter samples were used as negative control templates. Collected bees were preserved in RNA later, transported at RT and stored at –20 °C in the laboratory until DNA extraction.

### 2.2. Genomic DNA Extraction

*Aethina* samples were crushed individually in 2 mL micro centrifuge tubes containing 5 mm metal beads and 100 µL TN buffer (10 mmol/L Tris, 10 mmol/L NaCl; pH 7.6). Crushed samples were homogenized with a TissueLyser (QIAGEN, Hombrechtikon, Switzerland) for 25 s at 20 1/s frequency using a Qiagen Retsch^®^MM 300 mixer mill (Thermo Fisher Scientific, Zurich, Switzerland) and centrifuged for 2000 rpm. 50 µL of the supernatant was taken from the homogenate and used for DNA extraction using innuPrep DNA Mini Kit (Analytik Jena, Jena, Germany) by following the manufacturer’s recommendations. The DNA yield and purity of each sample were checked using a Spectrophotometer Thermo Scientific™ Nanodrop 2000 (Axon lab, Baden-Dättwil, Switzerland).

Prior to DNA extraction collected bee samples were carefully visually screened to ensure there are no SHBs (adults, larvae, and eggs) or beetle parts. Pooled bee samples (*N* = 100) from each colony were crushed in filter grinding bags (BIOREBA AG, Reinach, Switzerland) containing 20 mL TN buffer (10 mmol/L Tris, 10 mmol/L NaCl; pH 7.6) and homogenized with a homex6 (BIOREBA AG, Reinach, Switzerland). Aliquots of 50 μL of the homogenate in each case were used for DNA extraction using innuPrep DNA Mini Kit (Analytik Jena, Jena, Germany) by following the manufacturer’s recommendations. After DNA extraction, a spectrophotometer Thermo Scientific™ Nanodrop 2000 was used to quantify the DNA of each pooled sample.

### 2.3. Species-Specific PCR Assays

Eight SHB species-specific primer sets were developed using the exon 1 region of Cytochrome c oxidase subunit I (COI) genome (GenBank accession number: NW_017855158.1) with the online tool primer3Plus [12]. All primers were synthesized by Microsynth AG (Balgach, Switzerland). Designed primer sets were then tested with one sample each from all SHB populations and for the three other *Aethina* species. PCRs were carried out in 25 μL (15.88 μL millipore water, 5 μL 5× reaction buffer, 1 μL (0.4 μmol/L) each primer (reverse and forward), 0.125 µL (0.63 units) *Taq* DNA polymerase, 2 µL tenfold-diluted DNA) in a Biometra^®^ Thermal Cycler (Thermo Fisher Scientific, Switzerland) as follows: 95 °C for 2 min, 35 cycles of 95 °C for 20 s, 56 °C for 30 s, 72 °C for 30 s, and 72 °C for 2 min. Positive and negative controls were included in each PCR (DNA of a previously identified SHB specimen from Nigeria (based on both morphometrics and DNA sequence data or millipore water). Aliquots of the PCR products were electrophoresed on a 2 % (*w*/*v*) agarose gel. To resolve any ambiguities associated with a lack of amplification products, the universal primers LCO1490 (5′-GGTCAACAAATCATAAAGATATTGG-3′) and HCO2198 (5′-TAAACTTCAGGGTGACCAAAAAATCA-3′) were used to amplify the COI region [13]. The detection of an amplification product for LCO1490/HCO2198 confirmed the presence of amplifiable DNA and the absence of *Taq* DNA polymerase inhibitors. The primer sensitivity was determined with a PCR series using the same primer concentration, but decreasing DNA concentrations (100, 50, 25, 10, 5, 1, 0.5, 0.1, 0.01, 0.001 ng/μL).

### 2.4. Bees as Matrices for PCR-Based Diagnostics

SHB species-specific primers were tested in PCR runs for all extracted bee DNAs. Conventional PCRs were carried out in a volume of 25 μL containing 15.88 μL millipore water, 5 μL 5× reaction buffer, 1 μL (0.4 μmol/L) of each primer (reverse and forward), 0.125 μL (0.63 units) of *Taq* DNA polymerase and 2 μL DNA (ranging from 56 to 255 ng/μL). All reactions were carried out in a Biometra^®^ Thermal Cycler using the following cycling parameters: 95 °C for 2 min, followed by 35 cycles of 95 °C for 20 s, 56 °C for 30 s, 72 °C for 30 s, and 72 °C for 2 min. DNA of the identified SHB specimen from Nigeria (=positive control) and millipore water for molecular biology applications instead of DNA template (=negative control) were included in each PCR series. Aliquots of the PCR products (10 μL) were electrophoresed on a 2% (*w*/*v*) agarose gel. All the Swiss samples were also tested with lys-1 F (5′-ACCCGATAATTCAACGACGA-3′) and lys-1 R (5′CATTCGACCCTGGTTCATTT-3′) primers [14] to exclude false negative results. The sensitivity (*Se*) and specificity (*Sp*) of the visual screening and of the novel PCR method were calculated following [15]

(1) $$Se=\frac{A}{\left(A+B\right)}\times 100\;\mathrm{and}\;Sp=\frac{C}{\left(C+D\right)}\times 100$$

where *A* = number of true positives, *B* = number of false positives, *C* = number of true negatives, *D* = number of false negatives.

## 3. Results

### 3.1. Species-Specific PCR Assays

**Figure 1:**
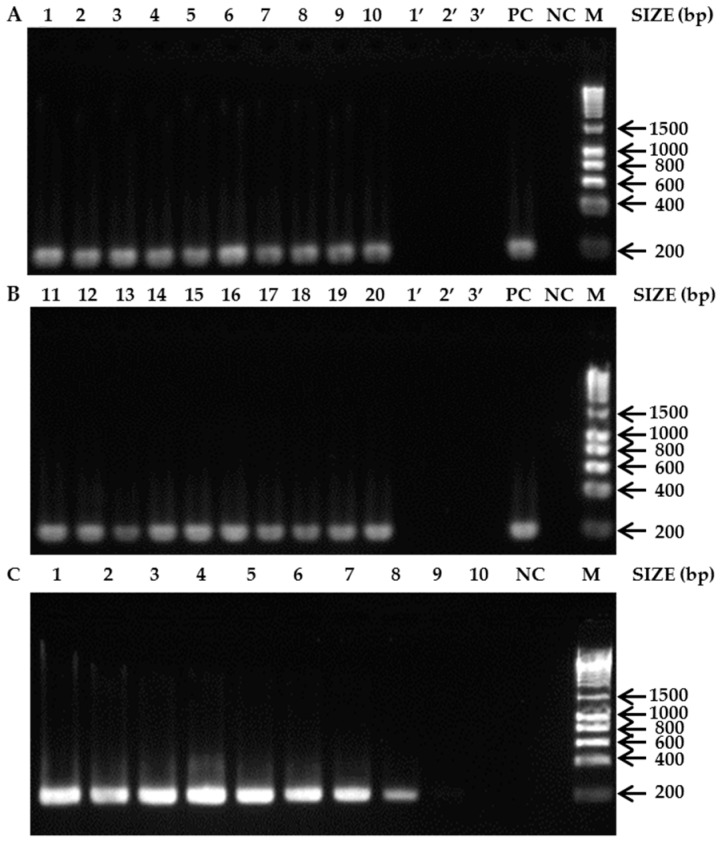
Agarose gels of PCR products. (**A**) *A. tumida* specimens from its native range and other *Aethina* spp: 1. *A. tumida* (Benin); 2. *A. tumida* (Togo); 3. *A. tumida* (Nigeria); 4. *A. tumida* (Central African Republic); 5. *A. tumida* (Burkina Faso); 6. *A. tumida* (Democratic Republic of Congo); 7. *A. tumida* (South Africa); 8. *A. tumida* (Ethiopia); 9. *A. tumida* (Tanzania); 10. *A. tumida* (Madagascar); (**B**) *A. tumida* specimens from its introduced ranges and other *Aethina* spp.: 11–12. *A. tumida* (US); 13. *A. tumida* (Brazil); 14. *A. tumida* (Costa-Rica); 15–16. *A. tumida* (Australia); 17. *A. tumida* (Portugal); 18. *A. tumida* (Italy); 19. *A. tumida* (Jamaica); 20. *A. tumida* (South Korea); 1’. *A. inconspicua* (South Korea); 2’. *A. flavicollis* (South Korea); 3’. *A. concolor* (Australia); (**C**) Gel of PCR validating the sensitivity test for the primer pair AT430F and AT622R. The concentration of template DNA from lane 1 to lane 10 was 100, 50, 25, 10, 5, 1, 0.5, 0.1, 0.01, 0.001 ng/µL respectively. PC = Positive control; NC = Negative control; M = Molecular marker (HyperLadder™ 1 kb).

From the eight primer sets developed, only one (AT430F (5′-GCTAAGTTAACTGAAGATCCACCAT-3′) and AT622R (5′-TAGTTCCACTAATACTAAGAGCCCC-3′)) proved to be reliable for SHB detection and identification, because all tested SHB individuals yielded amplification products of the expected size 193 bp (lanes 1–20, Figure 1A,B) and no amplification products were obtained for any of the non-target species: *A. concolor*, *A. flavicollis*, and *A. inconspicua* (lanes 1’–3’, Figure 1A,B). The successful amplification of products from all of these samples with the universal primers LCO1490/HCO2198 confirmed the presence of amplifiable DNA and the absence of *Taq* DNA polymerase inhibitors thereby validate the negative results (data not shown). PCR with the specific primer pair AT430F/AT622R and series of diluted crude DNA from a single SHB specimen were used to test the sensitivity. The primer pair yielded positive results for 0.1 ng/µL of the diluted SHB DNA (lane 8, Figure 1C).

### 3.2. Bees as Matrices for PCR-Based Diagnostics

**Figure 2:**
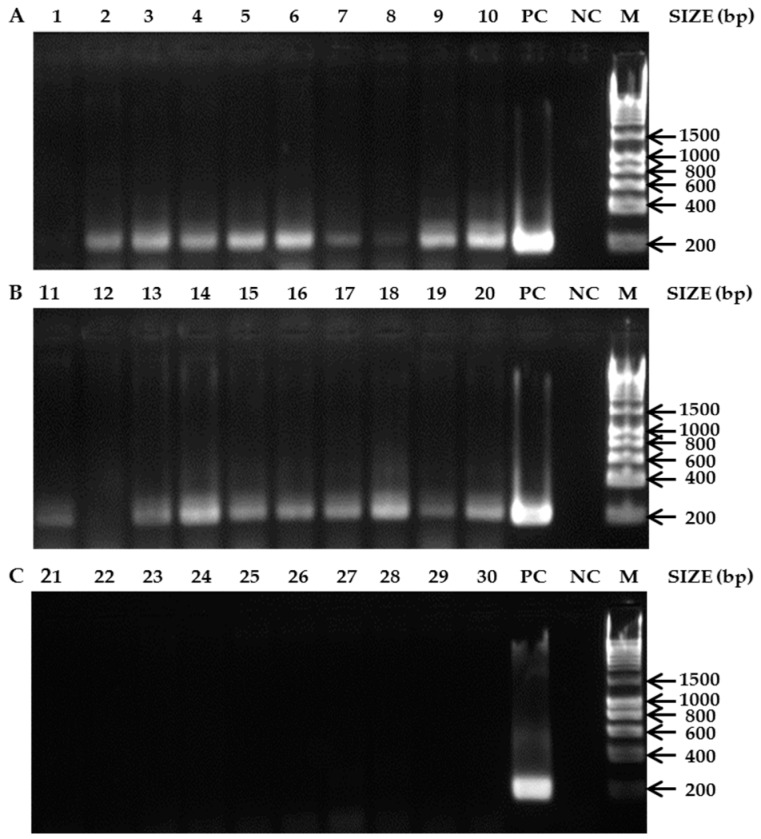
Agarose gels of PCR products. (**A**) Non-infested *A. mellifera* colonies from Nigeria based on visual screening; (**B**) SHB-infested *A. mellifera* colonies from Nigeria based on visual screening. (**C**) SHB-free *A. mellifera* colonies from Switzerland. 1. *A. mellifera* colonies (Ile-Ife); 2. *A. mellifera* colonies (Otan-Ile); 3. *A. mellifera* colonies (Lampese); 4. *A. mellifera* colonies (Logbara); 5. *A. mellifera* colonies (Tilden fulani); 6. *A. mellifera* colonies (Gubi); 7. *A. mellifera* colonies (Sarkin Pawa); 8. *A. mellifera* colonies (Kaltungo); 9. *A. mellifera* colonies (Kumo); 10. *A. mellifera* colonies (Rano); 11. *A. mellifera* colonies (Idi-Isin); 12. *A. mellifera* colonies (Ibule-Soro); 13. *A. mellifera* colonies (Ibule-Soro); 14. *A. mellifera* colonies (Ota); 15. *A. mellifera* colonies (Ota); 16. *A. mellifera* colonies (Logbara); 17. *A. mellifera* colonies (Logbara); 18. *A. mellifera* colonies (Itagbere); 19. *A. mellifera* colonies (Gubi); 20. *A. mellifera* colonies (Bebeji); 21. *A. mellifera* colonies (Aare 1); 22. *A. mellifera* colonies (Aare 2); 23. *A. mellifera* colonies (Aare 3); 24. *A. mellifera* colonies (Aare 4); 25. *A. mellifera* colonies (Liebefeld 1); 26. *A. mellifera* colonies (Liebefeld 2); 27. *A. mellifera* colonies (Liebefeld 3); 28. *A. mellifera* colonies (Liebefeld 4); 29. *A. mellifera* colonies (Liebefeld 5); 30. *A. mellifera* colonies (Liebefeld 6); PC = Positive control; NC = Negative control; M = Molecular marker (HyperLadder™ (Bioline Reagents Limited, London, UK) 1 kb).

The detection of an amplification product for the Lys-1 primers for each Swiss colony (data not shown) illustrates that the DNA quality was adequate and that the absence of a PCR product for the SHB primers was due to absence of *A. tumida* DNA. For the SHB primers the amplicons of the expected size were obtained: 193 bp (Figure 2A,B). From the 10 Nigerian colonies visually diagnosed as non-infested, 90 % of the colonies showed nevertheless positive PCR results (lanes 2–10, Figure 2A). Similarly, from the 10 Nigerian colonies visually diagnosed as infested, 9 colonies were tested positive with PCR (lanes 11, 13–20, Figure 2B). None of the 10 tested Swiss SHB-free colonies yielded any positive results (lanes 21–30, Figure 2C). The PCR diagnoses achieved a sensitivity of 100% and a specificity of 90.91%, compared with a sensitivity of 100% and a specificity of 52.63% for the visual screening.

## 4. Discussion

The data clearly show that the developed primers reliably detect SHB in all tested populations, even at low DNA concentrations and to separate it from three species of the same genus. Since rapid detection and reliable identification of SHB is crucial to limit further distribution of this species [8], these developed primers are expected to be valuable, rapid, reliable, and inexpensive tools for PCR diagnosis of this mandatory pest.

Our results also show that adult honey bee workers can be used as matrices for PCR-based detection of SHB’s DNA. The sensitivity of this novel PCR diagnostic method appears to be 100%, which is identical to conventional visual screenings [11]. Furthermore, the specificity of the PCR diagnosis (90.91%) was much higher compared to the visual screening (52.63%). This could be due to adult SHBs escaping during the inspections. Alternatively, but not mutually exclusive, SHBs may have simply left the colonies prior to inspections. Most likely, however, the low specificity of the visual screening in this particular case resulted from restriction to the bottom boards only, on which only about 50% of the adult SHBs can be found [1]. Due to a lack of respective data so far, it can obviously not be excluded that equipment contaminated with SHB DNA (e.g., introduced combs from infested other hives) may yield positive PCR results, thereby leading to false positive results. Given that this holds true, beekeepers should be advised not to recycle equipment from infested hives to facilitate diagnosis. Since both adult bees and adult SHBs frequently move between colonies within an apiary [16,17], it may also well be that bees from SHB-infested colonies enter non-infested ones, thereby leading to false positive PCR results. However, this appears less relevant at early invasion stages, where it seems rather important to catch the first infestation at an apiary level. Adult bees from outer frames are more likely to interact with SHBs [1]. We therefore suggest sampling adult bees from those frames too. Since both sensitivity and specificity are very high, this novel PCR method combined with the species-specific new primers can be expected to be a valuable and reliable tool for routine surveillance of hives in high-risk areas.
